# Accelerometry-Derived Sedentary Behavior & Physical Activity With Brain Structure: A Compositional Data Analysis

**DOI:** 10.1093/geroni/igaf122.1953

**Published:** 2025-12-31

**Authors:** Ryan Dougherty, Xinkai Zhou, Jill Rabinowitz, Lin Yee Chen, Vadim Zipunnikov, Adam Spira, Amal Wanigatunga, Jennifer Schrack

**Affiliations:** Rutgers University, New Brunswick, New Jersey, United States; Johns Hopkins University, Baltimore, Maryland, United States; Rutgers University, New Brunswick, New Jersey, United States; University of Minnesota School of Medicine, Minneapolis, Minnesota, United States; Johns Hopkins University, Baltimore, Maryland, United States; Johns Hopkins Bloomberg School of Public Health, Baltimore, Maryland, United States; Johns Hopkins Bloomberg School of Public Health, Baltimore, Maryland, United States; Johns Hopkins Bloomberg School of Public Health, Baltimore, Maryland, United States

## Abstract

Higher levels of physical activity and lower sedentary behavior in mid-to-late adulthood are each linked to better brain health outcomes, but their combined influence on brain structure remains unclear. We used compositional data analysis to investigate how the distribution of device-measured physical activity and sedentary behavior is associated with MRI measures of brain cortical thickness in 290 adults free of dementia (mean age 76.0±3.7 years, 60% women) from the ARIC study who wore a wrist accelerometer for one week and completed an MRI scan. Averaged-log-ratios between activity intensity categories (sedentary, low-light [lower-intensity light], high-light [higher-intensity light], moderate-to-vigorous) were constructed for compositional data analysis to examine covariate-adjusted associations with cortical thickness in the frontal and temporal lobes. There were significant associations with all log ratios of moderate-to-vigorous physical activity and temporal lobe cortical thickness, such that allocating more time to moderate-to-vigorous activity instead of sedentary, low-light, and high-light activity was associated with greater cortical thickness (ꞵrange=.019-.026, all FDR p < 0.05). Further, the low-light to high-light log ratio was significantly associated with greater temporal cortical thickness (ꞵ=.083, FDR p = 0.002). In contrast, there were no observed associations with frontal lobe cortical thickness (all FDR p > 0.05). These findings underscore the potential importance of physical activity intensity in maintaining brain cortical thickness, particularly in the temporal regions. Moderate-to-vigorous physical activity was linked to the best outcomes, but even slightly higher levels of activity intensity (i.e., low-light to high-light) showed potential neuroprotective associations. Longitudinal studies are needed to elucidate the temporality of the associations and biological mechanisms.

